# Flying with nut and other food allergies: unravelling fact from fiction

**DOI:** 10.1136/archdischild-2024-327848

**Published:** 2024-10-16

**Authors:** Paul Turner, Nigel Dowdall

**Affiliations:** 1 National Heart & Lung Institute, Imperial College London, London, UK; 2 Aviation Medical Consultancy Limited, Burgess Hill, UK

**Keywords:** Allergy, Allergy and Immunology, Child Health, Paediatrics

## Abstract

There is a common perception that peanut/tree nut particles can be transmitted through aircraft ventilation systems and pose a significant risk to passengers with food allergies. In fact, food-induced allergic reactions are around 10–100 times less common during flights than ‘on the ground’, perhaps because of the multiple precautions food-allergic passengers take when flying. We review the evidence for strategies to help prevent accidental allergic reactions while travelling on commercial flights (review registered at PROSPERO, ref CRD42022384341). Research studies (including aircraft simulations) show no evidence to support airborne transmission of nut allergens as a likely phenomenon. Announcements requesting ‘nut bans’ are not therefore supported, and may instal a false sense of security. The most effective measure is for passengers to wipe down their seat area (including tray table and seat-back entertainment system). Food proteins are often ‘sticky’ and adhere to these surfaces, from where they are easily transferred to a person’s hands and onto food that might be consumed. Airline companies can help to facilitate this through pre-boarding. Passengers at risk of anaphylaxis should be prescribed two adrenaline [epinephrine] autoinjector devices, to carry on their person at all times—including when flying. Airlines should consider including a separate supply of ‘general use’ adrenaline autoinjectors in the onboard medical kit for use in an emergency. All airlines should have clear policies relating to food allergies which are easily available from their websites or on request. These policies should be applied consistently by both ground staff and cabin crew, in order to provide reassurance to food-allergic passengers and their caregivers.

 Around 2–3% of children and 1–2% of adults in the UK have a food allergy,^1^ with similar prevalence in other medium–high income countries.^2^ Food allergy is the most common cause of anaphylaxis,^3^ a serious allergic reaction which can be life threatening. Fear over the potential for severe reactions results in a significant negative impact on quality of life. Vacations and travel pose a particular concern. In a global survey of 4704 food-allergic passengers and their caregivers, 98% reported increased anxiety when flying; high anxiety levels were reported by two-thirds of respondents.^4^ Over one-third reported unprofessional or insensitive behaviour from airport/airline staff. Reported problems ranged from home-made food being ‘ruined’ during routine airport inspections (in 25% of cases) to over 10% being asked to provide a medical note to verify the need to carry an adrenaline [epinephrine] autoinjector, with the devices sometimes being confiscated.^4^

There is a common perception that the risk of allergic reactions is increased when travelling by air^4 5^; however, a recent meta-analysis found that allergic reactions during commercial air travel are around 10–100 times less common than when ‘on the ground’ (figure 1).^6^ However, this needs to be interpreted in the context of the multiple precautions taken by food-allergic passengers when travelling, ranging from avoiding flying in the first place to bringing their own food to consume.^7^ This is likely to have an impact on actual risk. Disagreements with airline staff are not uncommon, and occasionally result in forced disembarkment (as evidenced by media reports). Airline policies with respect to food allergies are not always readily available,^8 9^ and can differ significantly between air carriers; policies may be implemented inconsistently by cabin crew and ground staff.^4 5 7^

**Figure 1:**
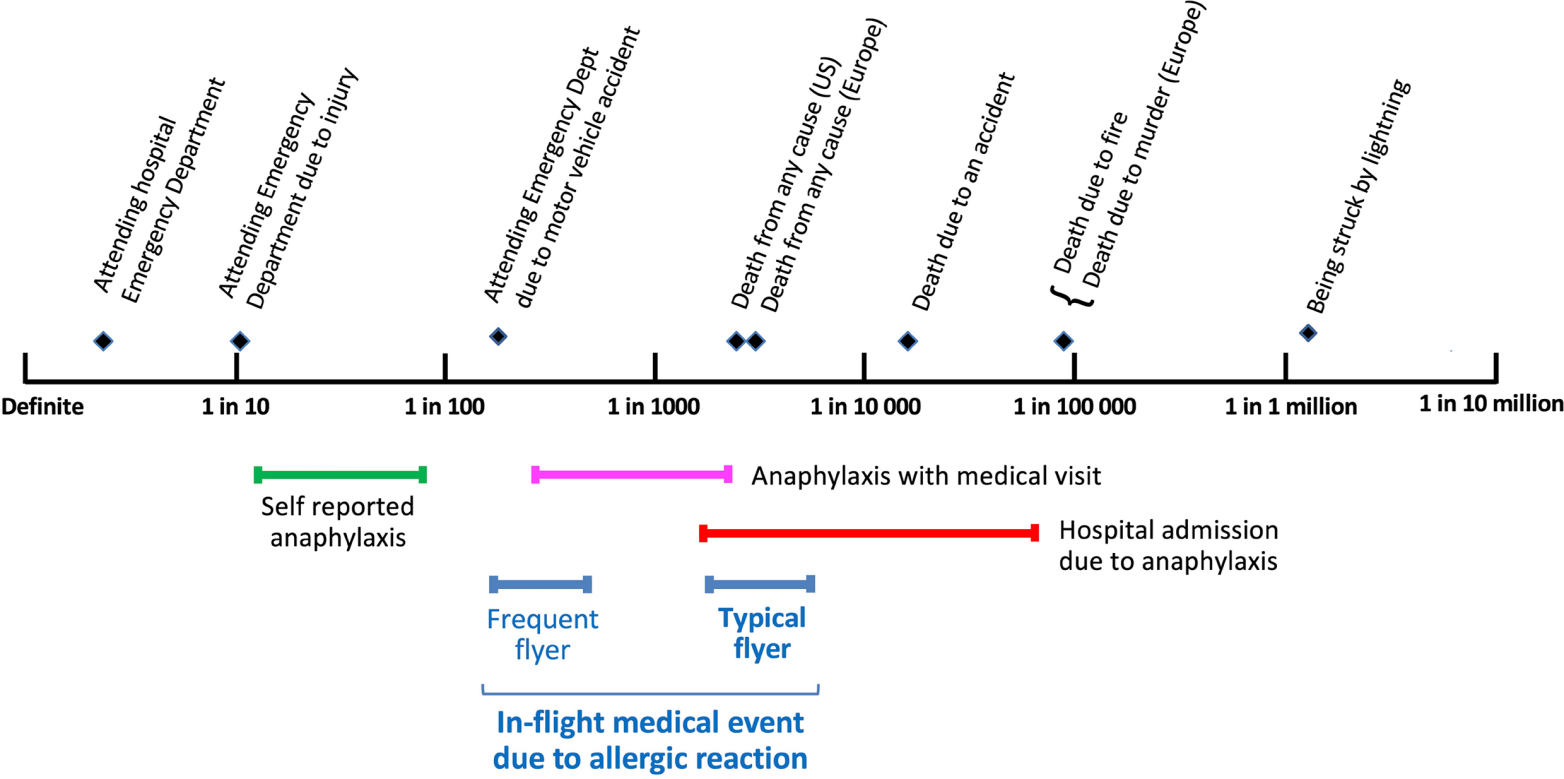
Estimated rates of food-induced allergic reactions in people with known food allergy during commercial flights (assuming a prevalence of 2% for food allergy) compared with equivalent rates when not flying and other risks.^6^ Data are shown as 95% confidence intervals. Reproduced with permission.

In 2023, the UK’s Civil Aviation Authority (CAA) commissioned a systematic review of the literature published from 1 January 1980 until 31 December 2022 relating to risks posed to food-allergic individuals on commercial flights, and how these might be mitigated. The review was registered with the International Prospective Register of Systematic Reviews (PROSPERO, reference CRD42022384341). We summarise the findings of the CAA report,^10^ highlighting some of the misconceptions which can hinder providing a safe flying environment for food-allergic individuals.

## Can food-allergic people react to aerosolised food particles?

Respiratory reactions to aerosolised food particles have been described in the literature (table 1),^11^,^16^ but such reactions are rarely reproducible.^13^ There are two important exceptions: people with allergy to fish/seafood often react to vapours from these foods (for example, due to a fish counter in a shop or cooking fumes).^14^ Many proteins in fish/seafood are volatile amines which are readily aerosolised at room temperature, and can therefore cause hay fever-like symptoms in the respiratory tract and occasionally, wheezing. Exposure to occupational allergens (eg, wheat flour in baker’s asthma, seafood in fish market workers) is another exception.^17^

**Table 1:** Studies reporting the incidence of allergic reactions due to potential, non-occupational inhalation of aerosolised food.

Study	Methodology	Population sampled	N	Food allergen	Proportion reporting reactions via inhaled route
Sicherer, 2001^11^	Registry, self-report	School-aged children	100 out of a cohort of 750 reporting reactions	Peanut, tree nuts	16 (16%)
Eigenmann, 2002^12^	Online survey	Age 1–61 years	51	All	3 (6%)
Roberts 2002^13^	Prospective study of clinic patients	Children	750	Fish, chickpea, cow’s milk, egg, buckwheat	12 (1.6%)
Turner, 2011^14^	Postal survey with telephone interview by trained HCP	Children/young people	167	Fish/seafood	26 (16%)
Fleischer, 2012^15^	Prospective observational study	Children aged3–15 months with possible food allergy	512, reporting1171 (unverified) reactions	Cow’s milk, egg, peanut	14/1171 reactions reported
Nguyen-Luu, 2012^16^	Retrospective clinic cohort	Children <18 years	1411, reporting266 reactions	Peanut	13 (0.9%)

HCP, healthcare professional.

There is a common perception that reactions due to aerosolised peanut are common, particularly on commercial aircraft; however, evidence suggests such instances are rare.^18^ Simonte *et al* recruited 30 peanut-allergic children (11 of whom reported previous inhalational reactions) who underwent a double-blind placebo-controlled inhalational challenge to peanut butter held 12 inches from the face for 10 min.^19^ None developed symptoms during the inhalation challenge (although one reported transient oral itch to placebo). Lovén Björkman *et al* performed an unblinded airborne peanut challenge (exposure to 300 g roasted peanuts in a bowl, placed approximately 50 cm in front of the patient in a small room) in 84 peanut-allergic children; only two developed mild symptoms (mild rhinoconjunctivitis, oral itch) and neither required treatment.^20^

**Figure 2:**
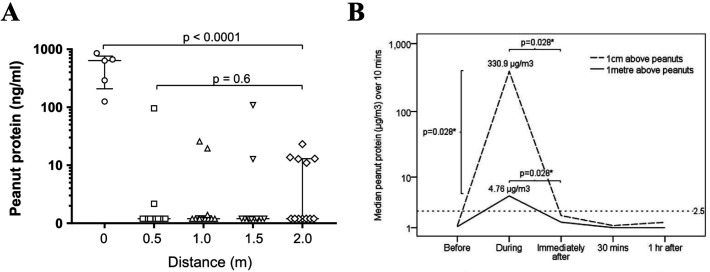
Detection of peanut in airborne samples. (**A**) After opening 200 g of roasted peanuts into a container and shaking them for 3 s, every 10 min, peanut could only be detected at significant level immediately above the open container.^20^ (**B**) Brough *et al* assessed airborne peanut during peanut deshelling. Air sampling was performed for 10 min before, during, immediately after, 30 and 60 min after deshelling peanuts at 1 cm and 1 m above the peanuts. Peanut was only detected during actual deshelling, and not afterwards.^22 23^ Reproduced under a Creative Commons CC-BY-NC-ND 4.0 International licence.

Arguably, deshelling roasted peanut is the most likely scenario that might result in aerosolised peanut allergen. Studies have shown that deshelling can result in very low level but detectable peanut allergen in the air directly above the peanuts (figure 2)—but only briefly during actual deshelling—implying that the peanut dust is like to settle and not circulate in the air under normal conditions.^20^,^23^ These data are consistent with those of Perry *et al*, who were unable to detect airborne peanut allergen in simulated real-life situations when participants consumed peanut butter, shelled and unshelled peanuts, including in a confined space to simulate an aircraft cabin.^24^

### Summary

With a few notable exceptions (eg, fish/seafood, occupational wheat allergy), reactions to aerosolised foods are very uncommon and rarely reproducible. Peanut allergens can be detected at very low levels in the air when shelling nuts, but the dust settles quickly and can only be detected in very close proximity to the nuts.

## Can food allergens be spread through aircraft cabin ventilation systems?

**Figure 3:**
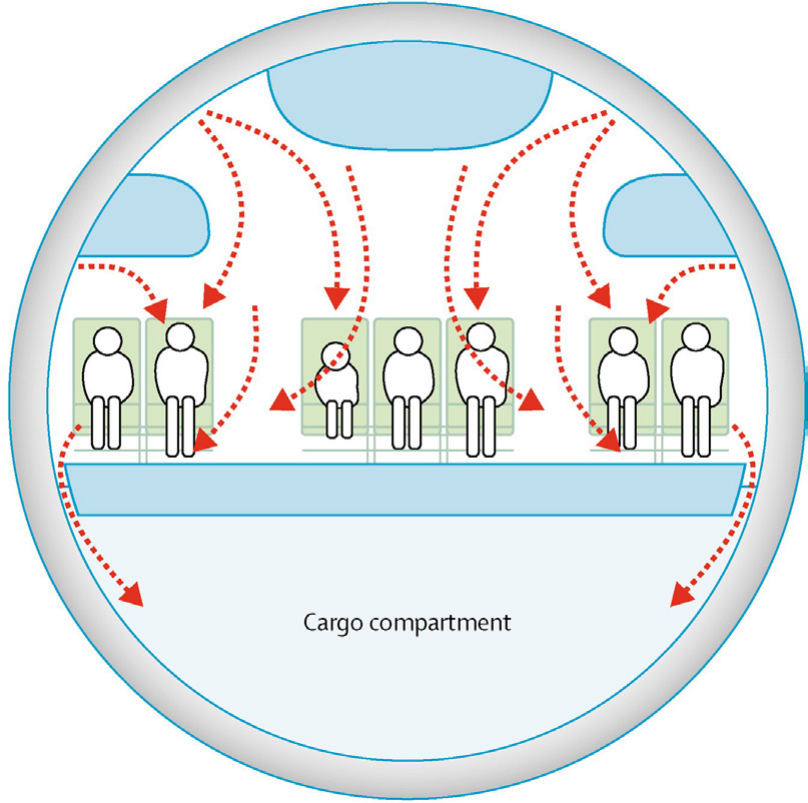
Model of air circulation in a passenger cabin on commercial aircraft.^25^ Copyright 2005 Elsevier Ltd. Re-use granted by Elsevier as part of the Elsevier COVID-19 resource centre.

To provide a safe and comfortable environment for staff and passengers, aircraft have environmental control systems (ECS) which manage cabin air pressure, air supply and temperature. ECS must also ensure adequate removal of carbon dioxide, odours and other airborne contaminants (including pathogens), which requires high airflow rates within the cabin. Adequate ventilation is achieved by air being supplied into the cabin through overhead distribution outlets which run the length of the cabin. The system is designed to create a controlled circular pattern of airflow, with air continuously extracted through vents at floor level. This results in air circulating *across* the aircraft, rather than *along* the cabin (figure 3), which minimises the potential for spreading passenger-generated contaminants through the passenger cabin. Typically, ECS are designed to provide approximately 20 cubic feet (566 litres) of air per minute per passenger, resulting in a complete cabin air exchange every 3–4 min.^25^ For comparison, air in hospital rooms and classrooms is exchanged about every 10 min. In modern large commercial aircraft, around half of the air intake is recirculated air which has passed through high efficiency particulate air (HEPA) filters (the other 50% of air supply comes from outside the aircraft). The HEPA filters used on commercial aircraft have a particle-removing efficiency of 99·97% at 0·3 µm, which effectively remove dust, vapours, potential microbial pathogens, and capture the vast majority of aerosolised food particles at the same time. For example, typical particle sizes for peanut dust range from 2 to 30 µm^26^ – so HEPA units (which have a particle-removing efficiency of 99·97% at 0·3 µm) would prevent recirculation of any peanut dust into the air cabin.

Consistent with these data, Jones *et al* detected peanut in eluent from filter units from two commercial aircraft at the time of their annual replacement (after approximately 5000 flight hours).^27^ Paciencia *et al* analysed dust collected from the cabin carpet and seats on 10 short-haul and medium-haul commercial airplanes, as part of routine aircraft cleaning.^28^ Peanut was detected in all samples analysed, up to a maximum of ~600 mg total peanut protein per gram of dust. Typical estimates for (unintended) dust consumption are around 100 mg dust per day. Therefore, an exposure equivalent to 6 hours (typical of a medium-haul flight) could in theory result in an exposure sufficient to trigger subjective symptoms in 30–50% and objective allergic symptoms in 10% of peanut-allergic individuals.^29^ However, this assumes that a passenger would be ‘fully exposed’ to peanut residue in dust suspended in the cabin air for the entire duration of the flight—something which cannot occur given the efficiency of HEPA filtration and the frequency of complete cabin air exchange (15–20 times an hour).

Studies have demonstrated that peanut is easily transmitted, both through touch and in saliva.^22 23^ Jin *et al* measured peanut present in surface swabs from aeroplane tray tables and seats and air samples, taken during a commercial flight in which deshelled roasted peanuts were eaten, and another flight when no peanuts were served.^30^ Peanut protein was found in swabs taken from both the tray table and seat irrespective of whether peanut was served. The highest amounts were found in swabs taken shortly after eating peanut (table 2). No peanut was detected in air samples taken away from the site of peanut consumption; only one air sample, collected during active peanut consumption at the level of the tray table, had very low level peanut. The authors conclude that ‘any potential for accidental exposure to peanut protein in airplanes stems from surface contamination, not airborne exposure.’ These data explain the discrepancy between the perception that ‘airborne peanut’ is a common cause of allergic reactions, and study data demonstrating an extremely low risk of reaction due to aerosolised peanut in challenge studies.

**Table 2:** Detection of peanut during active flights, as reported by Jin *et al*^30^

Surface sampled	Timing	Peanut served?	Estimated peanut protein (mg) per square foot
Seat and tray	During boarding (prior to peanuts being served)	Yes(after sampling)	21.5
Seat and tray	During boarding (prior to peanuts being served)	Yes(after sampling)	3.1
Seat and tray	Mid-flight (immediately after peanuts eaten)	Yes	441
Seat and tray	During boarding	No	1.2
Tray	Mid-flight	No	6.2 - 16.1

### Summary

Studies show that:

1. Peanut residue can be found on aircraft surfaces (seats, trays, floor), usually due to consumption of peanut on prior flights.
2. Any peanut particles which might be present in the air would not be spread through the cabin but enter the ECS and be removed by the HEPA filtration system, thus posing negligible risk to peanut-allergic individuals.
3. Peanut residue present on aircraft surfaces (seats, seat-back entertainment systems, trays) may be transmitted to the hands and then transferred either to food being consumed or directly to the individuals mouth/face.

## Are ‘nut bans’ effective on board aircraft?

Given the above findings, announcements asking all passengers to refrain from eating nuts are unlikely to be effective in protecting nut-allergic passengers, since the amount of peanut residue present in the aircraft cabin will mostly depend on whether peanut was eaten on previous flights. Unless aircraft cabins are *always* nut-free, there might be nut residue present, but at levels that pose negligible risk in terms of airborne transmission.

## What strategies may be effective to reduce the risk of allergic reactions?

Studies have looked at the efficacy of different environmental measures in removing surface peanut residue. Detergents (including wet-wipes and handwashing with soap) are more effective than water alone or alcohol-gel based agents.^23 24^ Cleaning tray tables, seat surfaces and seat-back entertainment systems at the start of a flight using cleaning or sanitising wipes is therefore likely to be effective in reducing the risk posed by residual food proteins to food-allergic passengers.^7^ This is particularly important, given the minimal cabin cleaning which occurs in between flights, especially with low-cost carriers.

Whether ‘buffer zones—’where passengers travelling in the immediate vicinity of a food-allergic passenger are asked not to consume the relevant allergen—can reduce risk is unclear. In theory, consumption of some allergic foods (such as nuts) immediately next to a food-allergic passengers might pose a small degree of risk due to airborne particles which are deposited (on food or surfaces) before being extracted through the ECS. More research is needed to assess this risk, and whether ‘buffer zones’ can reduce this (and how this might depend on the size of the exclusion zone). Notwithstanding, ‘buffer zones’ are likely to provide reassurance to food-allergic passengers, and avoid the scenario whereby a food-allergic passenger is seated next to another passenger consuming the food to which they are allergic.^5^ Implementing ‘buffer zones’ also raises the question of which allergens other passengers can be reasonably requested not to consume. Passengers also should note that such measures will not reduce any allergen residue presence on other surfaces in the aircraft that they might touch (eg, bathroom door handles).

Many food-allergic passengers bring their own food to eat while flying as a precaution. This might be made at home, but increasingly, food-allergic people purchase what they perceive to be ‘safe food’ from airport outlets. In a prospective study of 498 children over 1 year, five in-flight allergic events were reported: three were due to consumption of the allergen in the food purchased as a ‘safe’ alternative prior to boarding, and one to a home-made sandwich.^31^ This highlights the risk of human error in preparing for travel.

### Summary

In addition to dietary avoidance of trigger allergens, cleaning the seat area (including the tray table and the seat-back entertainment system) is likely to be the most effective measure food-allergic passengers can take to mitigate against the risk of unintended allergic reactions.

## Should aircraft carry adrenaline autoinjectors?

The International Civil Aviation Organization provides recommendations on the provision of first-aid training for cabin crew and contents of first aid and onboard medical kits.^32^ National aviation authorities specify detailed regulations with which airlines are required to comply: these often include adrenaline in the onboard medical kit as stock vials.^33 34^ There are no specific requirements for carriage of adrenaline autoinjectors (although many larger international airlines choose to include these in the medical kit). It is important that healthcare professionals and travellers at risk of anaphylaxis do not automatically assume that adrenaline will be available in an emergency. Passengers at risk of anaphylaxis and who have been prescribed adrenaline autoinjectors must therefore travel with these in the cabin when flying (and not checked into hold luggage).^35^ It should not be assumed that cabin crew will help to administer adrenaline in an emergency, although cabin crew (where permitted by national regulations) may be allowed to administer it to an individual experiencing anaphylaxis.

## Discussion

There is a common misconception that allergic reactions can occur due to inhalation of aerosolised peanut and tree nut particles, and any risk can be mitigated by requesting all other passengers not to consume nuts during the flight. Food-allergic passengers (and those caring for them) need to be informed that the main risks are due to either accidental consumption of a trigger food, and to surface allergen residues which can then be transferred onto food or by direct hand-to-mouth/face inoculation. Simple strategies, including wiping down the seat area, seat table and in-flight entertainment system, appear to be effective in reducing the risk. Allowing food-allergic passengers to pre-board may be helpful (the US Department of Transportation already requires airlines to allow passengers with peanut/tree nut allergies to pre-board, if requested).^36^

**Figure 4:**
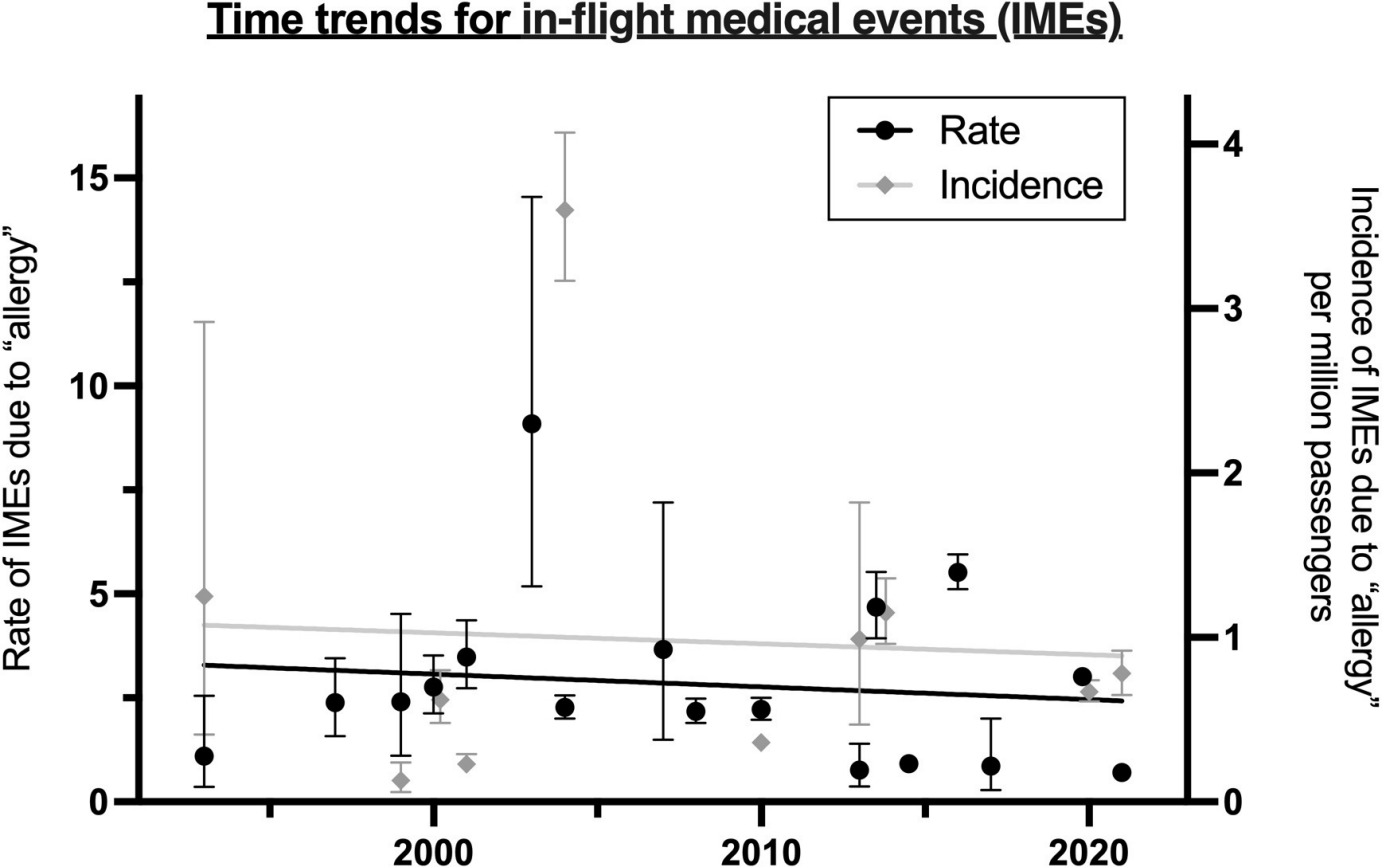
Time trends for in-flight medical events (IMEs) due to allergic reactions over the past two decades.^6^ Reproduced with permission.

Arguably, if peanuts/tree nuts are not provided with in-flight service, then there would be a lower risk to passengers with allergies to these foods. However, this may not be a valid assumption: at least one study has reported no difference in peanut present in household dust between homes where peanut was completely avoided (owing to allergic individuals in the household) and homes that did not restrict peanut.^37^ Furthermore, the rate of in-flight allergic reactions has not significantly changed over the past 30 years (figure 4),^6^ despite a significant drop in the number of airlines serving peanuts (although with the growth in low-cost short haul flights, it is likely that nut-based snacks are still consumed by many passengers during flights).

There is a concern that announcements requesting passengers *not* to consume a specific food might give a false sense of reassurance (as might be the case with ‘nut bans’ in schools),^38^ and increase the risk of confrontation among passengers and with cabin staff if someone does not comply with the request. As with schools, it is difficult to implement ‘bans’ to non-nut allergens (such as cow’s milk, egg, wheat or fish) if a passenger allergic to these foods is travelling. Some might assert that peanuts/tree nuts are of greater concern, but recent data show that cow’s milk is as common a cause of severe and fatal anaphylaxis as peanuts.^3^ Rather, there needs to be a focus on reducing risk due to allergen residues on surfaces, and airlines should have policies in place to facilitate this. While evidence in support of ‘buffer zones’ is currently lacking, such an approach is likely to provide important reassurance to food-allergic passengers and avoid the scenario whereby a food-allergic passenger is seated next to another passenger consuming the allergen to which they are allergic.^5^

Food-allergic individuals at risk of anaphylaxis should be prescribed two adrenaline autoinjector devices which they should carry on their person at all times, including when on board aircraft. While national aviation authorities typically require adrenaline ampoules (at the relevant concentration to treat anaphylaxis) to be carried as stock vials in the onboard medical kit, airlines should consider including a separate supply of adrenaline autoinjectors in the medical kit for cabin crew to use in an emergency, since their use requires minimal training. In the UK setting, this is very likely to be a cost-effective measure.^39^

## Conclusions

In this review, we summarise the evidence for or against various perceptions relating to flying with food allergies (table 3). There is no evidence that peanut or tree nut allergens are spread through aircraft cabin ventilation systems. Rather, the main risks are due to either failure of dietary avoidance, or allergen residues on surfaces, which can then be transferred through touch—a situation exacerbated by the very short turnaround times with many low-cost carriers. Therefore, announcements requesting passengers not to consume nuts during the flight are unlikely to be effective in reducing the risk of in-flight reactions, and might provide false reassurance. Wiping down the seat area (including seat table and seat-back entertainment system) using a wet wipe appears to be an effective strategy. Airlines should have clear policies relating to food allergies which are easily available from their websites or on request. These policies should be applied consistently by both ground staff and cabin crew, in order to provide reassurance to food-allergic passengers and their caregivers.

**Table 3:** Common misconceptions about flying with food allergies

Common ‘myths’	What evidence tells us
Myth 1: Allergic reactions to food are more common in the air than ‘on the ground’	Data show that food-allergic people are around 10–100 times less likely to have a reaction on an aeroplane. However, this might in part be due to the precautions they take when flying
Myth 2: People with food allergies commonly react to aerosolised food allergens	With a few notable exceptions (eg, fish/seafood, baker’s asthma), reactions to aerosolised foods are very uncommon and rarely reproducible
Myth 3: Nut particles can be transmitted through the aircraft cabin ventilation system and cause reactions	Airborne peanut can only be detected at very low levels when shelling nuts, and only very close to the nuts; any dust settles quickly. Aircraft cabin ventilation systems are designed to remove air *across* the cabin rather than along it. There is a complete air exchange in the cabin every 3–4 min, and filters capture >99.97% of any circulating nut particles.The more likely cause of accidental reactions is either due to: Accidental consumption of a food containing the allergen Transmission of allergen residue from seat/tray surfaces (including seat-back screens) to a person’s hands, which are then transferred to ‘safe’ food being consumed
Myth 4: Nut bans are effective	Announcements asking passengers to refrain from eating nuts are unlikely to be effective. There is far greater exposure from peanut residue left on seat surfaces etc from previous flights, than sporadic nut consumption during a flight. Cleaning tray tables, seat surfaces and seat-back entertainment systems at the start of a flight is much more likely to be effective at reducing risk. Nut ‘bans’ can also cause a false sense of security.
Myth 5: Avoiding aeroplane food is important	Many food-allergic passengers bring their own food to eat while flying. However, in-flight allergic reactions have been reported to both home-made food and food items purchased before boarding, at the airport, with human error resulting in the purchase of food/ingredients containing the trigger food. Where there is a meal service, most airlines offer allergen-free options if requested in advance.
Myth 6: A medical note is required to carry adrenaline autoinjectors on board aircraft	Under UK law, medical authorisation is not needed for individuals who are prescribed autoinjector devices to carry these during air travel. Note that autoinjectors are not often included in onboard medical kits, nor are cabin crew always allowed to use them. Therefore, food-allergic individuals at risk of anaphylaxis should be prescribed two adrenaline autoinjectors, which they should carry on their person when flying (and not checked into luggage).
